# The interplay between α-synuclein aggregation and necroptosis in Parkinson’s disease: a spatiotemporal perspective

**DOI:** 10.3389/fnins.2025.1567445

**Published:** 2025-04-08

**Authors:** Haoran Xiang

**Affiliations:** ^1^The First College of Clinical Medical Science, China Three Gorges University, Yichang, Hubei, China; ^2^Department of Neurology, Yichang Central People’s Hospital, Yichang, Hubei, China

**Keywords:** Parkinson’s disease, alpha-synuclein, necroptosis, mitochondrial dysfunction, oxidative stress, neuroinflammation, therapeutic strategies

## Abstract

Parkinson’s disease (PD) is a common neurodegenerative disorder characterized by the death of dopaminergic neurons and the aggregation of alpha-synuclein (α-Syn). It presents with prominent motor symptoms, and by the time of diagnosis, a significant number of neurons have already been lost. Current medications can only alleviate symptoms but cannot halt disease progression. Studies have confirmed that both dopaminergic neuronal loss and α-Syn aggregation are associated with necroptosis mechanisms. Necroptosis, a regulated form of cell death, has been recognized as an underexplored hotspot in PD pathogenesis research. In this review, we propose a spatiotemporal model of PD progression, highlighting the interactions between α-Syn aggregation, mitochondrial dysfunction, oxidative stress, neuroinflammation and necroptosis. These processes not only drive motor symptoms but also contribute to early non-motor symptoms, offering insights into potential diagnostic markers. Finally, we touch upon the therapeutic potential of necroptosis inhibition in enhancing current PD treatments, such as L-Dopa. This review aims to provide a new perspective on the pathogenesis of PD and to identify avenues for the development of more effective therapeutic strategies.

## 1 Background

Parkinson’s disease is a neurodegenerative disorder characterized by the death of dopaminergic neurons and the accumulation of alpha-synuclein (α-Syn). Necroptosis is a form of programmed cell death mediated by a signaling complex consisting of receptor-interacting protein kinase 1 (RIPK1), receptor-interacting protein kinase 3 (RIPK3), and mixed lineage kinase domain-like (MLKL) proteins (Wu et al., 2014). The occurrence of this type of cell death has been observed in *in vitro* and *in vivo* models of PD, as well as in brain tissue from PD patients (Iannielli et al., 2018; Oñate et al., 2020; Wu et al., 2015). Studies in mouse models have shown that inhibition of key proteins involved in necroptosis reduces dopaminergic neuronal degeneration and improves motor function (Iannielli et al., 2018). The necroptosis pathway, involving RIPK1-RIPK3-MLKL, has been definitively implicated in the progression of PD (Iannielli et al., 2018; Oñate et al., 2020). Moreover, α-Syn aggregation plays a central role in initiating and perpetuating PD pathology. It disrupts mitochondrial function, induces oxidative stress, and triggers neuroinflammatory responses, all of which can activate necroptosis. This creates a self-reinforcing cycle where necroptosis and α-Syn aggregation amplify each other, accelerating neuronal loss. In this review, we present a spatiotemporal model to illustrate how these pathological processes interact over the course of PD. By integrating mechanisms such as α-Syn aggregation, mitochondrial dysfunction, neuroinflammation, and necroptosis, we aim to provide a comprehensive framework for understanding disease progression. Additionally, we discuss the implications of targeting necroptosis as a therapeutic strategy and the potential benefits of combining this approach with existing treatments. This perspective underscores the need for innovative approaches to mitigate the devastating impact of PD on patients and society.

## 2 What is necroptosis?

**FIGURE 1:**
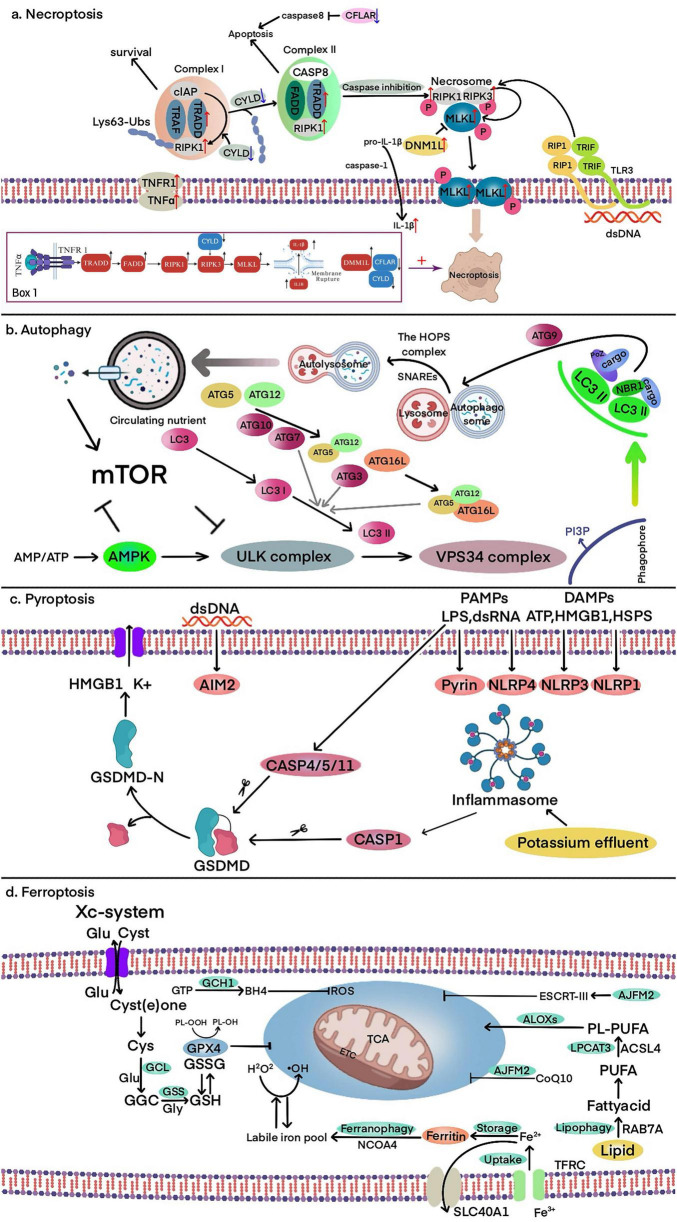
Core molecular mechanisms of autophagy, pyroptosis, ferroptosis, and necroptosis. **(a)** Necroptosis: TNFα binding to TNFR1 forms Complex I, where RIP1 polyubiquitination by cIAP promotes cell survival. Deubiquitination of RIP1 by CYLD facilitates the formation of Complex II, leading to CASP8 activation and apoptosis. When CASP8 is inhibited, RIPK1, RIPK3, and MLKL form the necrosome, activating MLKL and inducing necroptosis via membrane pore formation. Box 1. The core pathway and key molecular changes of necroptosis in PD. These molecular changes collectively enhance necroptosis directly or indirectly. **(b)** Autophagy: the ULK complex is activated in response to nutrient and energy stress, initiating VPS34-dependent PI3P production and recruitment of ubiquitin-like conjugation systems. LC3 lipidation facilitates cargo receptor recruitment (e.g., SQSTM1/P62), enabling autophagosome formation. The autophagosome expands with ATG9 and fuses with lysosomes, forming autolysosomes where degradation and recycling occur. **(c)** Pyroptosis: cytoplasmic sensors (e.g., NLRP1, NLRP3, AIM2) recognize PAMPs/DAMPs, activating CASP1 via ASC. CASP4/5/11 directly recognize LPS, leading to GSDMD cleavage. The N-terminal fragment of GSDMD forms membrane pores, triggering pyroptosis, potassium efflux, and the release of HMGB1 and other inflammatory mediators. **(d)** Ferroptosis: iron overload and lipid peroxidation drive ferroptosis. The TF-TFRC complex and ferritinophagy facilitate iron accumulation, while the ACSL4-LPCAT3-ALOX pathway promotes lipid peroxidation. Antioxidant defenses, including the Xc- system, GSH, and GPX4, counteract oxidative stress to prevent ferroptotic cell death.

Necroptosis is a form of receptor-mediated cell death, discovered in 2005 by the renowned scholar Professor Jun ying Yuan. It is characterized by a highly regulated process that does not rely on cysteinyl aspartate-specific proteinase (Caspase) proteases and exhibits typical necrotic morphological features (Degterev et al., 2005). In 2017, a research team led by Professor Salvatore Oddo from Arizona State University published a study in Nature Neuroscience. They discovered a new pathway of neuronal death, which turned out to be “necroptosis” (Caccamo et al., 2017). Necroptosis can be initiated by the activation of death receptors (DRs), such as Tumor Necrosis Factor Receptor 1 (TNFR1). Under pathological conditions, TNFR1 activation leads to the activation of Receptor-Interacting Protein Kinase 1 (RIPK1). In the absence of Caspase 8 (CASP8) activity, deubiquitinated RIPK1 interacts with RIPK3 through the RIP Homotypic Interaction Motif (RHIM), recruiting RIPK3. Mixed Lineage Kinase Domain-Like (MLKL) is then recruited by RIPK3, forming the RIPK1-RIPK3-MLKL complex (also known as Complex IIb) (Yuan et al., 2019). In the absence of Caspase 8 (CASP8) activity, deubiquitinated RIPK1 interacts with RIPK3 through the RIP Homotypic Interaction Motif (RHIM), recruiting RIPK3. RIPK3 undergoes autophosphorylation, forming a complex known as the necrosome. Within this complex, RIPK3 phosphorylates Mixed Lineage Kinase Domain-Like (MLKL), leading to the formation of active oligomers. These active MLKL oligomers disrupt the plasma membrane, causing cell lysis and ultimately triggering necroptosis (Shan et al., 2018; Weinlich et al., 2017). Increased levels of necroptosis biomarkers have been observed in patients with amyotrophic lateral sclerosis (ALS) and COVID-19 pneumonia (Ito et al., 2016; Ruskowski et al., 2022). A clinical trial has been conducted using RIPK1 inhibitors to treat COVID-19 infected patients (Nyberg et al., 2022). In the research conducted by our team, it was found that “Primidone,” a RIPK1 inhibitor, could delay the onset of motor deficits in the ALS animal model, the SOD1 G93A transgenic mice. It improved neurological scores and reduced weight loss. Additionally, from the perspective of “drug repurposing,” a clinical trial was carried out, revealing that “Primidone” could reduce the levels of necroptosis biomarker RIPK1 and the inflammatory cytokine interleukin-8 (IL-8) in ALS patients (Wei et al., 2023). Based on the above characteristics, we can distinguish necroptosis from other mechanisms of programmed cell death, such as autophagy, pyroptosis, and ferroptosis (Figure 1).

### 2.1 Necroptosis in PD

Compared to other forms of cell death, such as apoptosis or autophagic cell death, necroptosis in PD has distinct characteristics. It not only leads to membrane rupture, DAMP release, and neuroinflammation through the RIPK1/RIPK3/MLKL signaling pathway (Awasthi et al., 2025; Fricker et al., 2018; Levy et al., 2009; Liu et al., 2018; Mansour et al., 2023; Michel et al., 2016; Moujalled et al., 2021; Panicker et al., 2021; Venderova and Park, 2012), but also interacts closely with ferroptosis in a complex manner. In a study on ischemic stroke reperfusion, Du et al. (2024) found that necroptosis and ferroptosis can promote each other. Ferroptosis indirectly triggers necroptosis by promoting iron accumulation and oxidative stress. In turn, necroptosis leads to membrane rupture and the release of damage-associated molecular patterns (DAMPs), which intensify neuroinflammation and further contribute to iron accumulation through oxidative stress. This interplay creates a feedback loop in which ferroptosis and necroptosis mutually reinforce each other, potentially driving disease progression (Eigenbrod et al., 2008; Fischbacher et al., 2017; Johnson et al., 2019). These characteristics suggest that necroptosis may drive the progression of PD. A bioinformatics study identified several differentially expressed genes associated with necroptosis and proposed a framework for understanding the role of necroptosis in PD (Lin et al., 2023). The study found that RIPK1, RIPK3, and MLKL, as core signaling molecules of this pathway, exhibit altered expression and activity in the brain tissue of PD patients. Additionally, TRADD, a key component of the TNFR1 complex, was found to be abnormally expressed in PD, while CFLAR may help regulate the balance between necroptosis and apoptosis by modulating Caspase-8 activity. Furthermore, downregulation of CYLD may affect the deubiquitination of RIPK1, thereby enhancing the stability of the necroptotic signaling pathway. The study also observed that upregulation of DNM1L may contribute to mitochondrial dysfunction, exacerbating neuronal damage. DNM1L itself also interacts with core necroptosis factors such as MLKL, suggesting that it may play a role in PD-related necroptosis. IL1B, as a pro-inflammatory factor, showed increased expression in PD tissues, potentially amplifying local inflammatory responses. Building on these findings, we can propose a model (Box 1 in part a of Figure 1) in which RIPK1, RIPK3, and MLKL act as core effectors of necroptosis, while TRADD, CFLAR, and CYLD modulate this pathway in Parkinson’s disease. Additionally, DNM1L-mediated mitochondrial dysfunction and IL1B-driven neuroinflammation further amplify necroptotic signaling, exacerbating neuronal damage. This model provides a theoretical basis for understanding the molecular interactions underlying necroptosis in PD and offers potential targets for therapeutic intervention.

## 3 The two key pathological features of PD and necroptosis

### 3.1 Dopaminergic neuron death and necroptosis

In studies on necroptosis, several findings suggest that it plays an important role in dopaminergic neuron death. Wu et al. (2015) discovered that necroptosis occurred in PC12 cells treated with 6-hydroxydopamine (6-OHDA). Roy et al. (2023) observed that exposure to Rotenone in N2A cells significantly increased the levels of TNF-α (Tumor Necrosis Factor-α), along with phosphorylation of RIPK1, RIPK3, and MLKL, which are recognized as markers of necroptosis. Furthermore, studies have shown that dopaminergic toxins induce dopaminergic neuron death through different pathways. For example, 6-OHDA induces caspase-dependent cell death, MPP+ stimulates caspase-independent cell death, and rotenone activates both pathways (Callizot et al., 2019). Oñate et al. (2020) further discovered the activation of necroptosis in postmortem brain tissue from PD patients and in toxin-based disease mouse models. In mice treated with MPTP and in the brains of PD patients, necroptosis activation in the substantia nigra was observed, along with the loss of dopaminergic neurons. Additionally, the lack of miR-425 was found to be associated with this process. miR-425 targets the RIPK1 transcript and promotes the phosphorylation of MLKL, facilitating necroptosis (Hu et al., 2019). These studies collectively highlight the potential role of necroptosis in dopaminergic cell death, providing crucial insights for further research into Parkinson’s disease and related disorders.

In studies on the pathological mechanisms of PD, multiple experiments have demonstrated that necroptosis plays an important role. Pathological axonal degeneration is often observed in patients with neurodegenerative diseases (such as Amyotrophic Lateral Sclerosis, Alzheimer’s Disease, Multiple Sclerosis, and PD), which significantly contributes to neurological disability (Adalbert and Coleman, 2013; Conforti et al., 2014; Zhou et al., 2017). Axonal degeneration may result from direct nerve transection, known as Wallerian degeneration (Raff et al., 2002; Waller, 1851; Wang et al., 2012). Growing evidence indicates that oligodendrocyte degeneration and dysfunction are critical mechanisms driving axonal degeneration (e.g., Wallerian-like degeneration) in neurodegenerative diseases (Vargas and Barres, 2007). In a PD mouse model, Oñate et al. (2020) experimentally demonstrated that axonal degeneration in PD is mediated by necroptosis, referred to as necroptotic axonal degeneration. By inhibiting key components of the necroptosis pathway—such as genetic ablation of necroptosis mediators MLKL and RIPK3, or pharmacological inhibition of RIPK1—the degeneration of dopaminergic neurons was reduced, leading to improvements in motor function in the model (Oñate et al., 2020). Wu et al. (2015) used 6-OHDA to induce necroptosis in PC12 cells and also found that treatment with the necroptosis inhibitor Nec-1 (Necrostatin-1) could protect cell viability. Another study found that using a specific inhibitor of MLKL phosphorylation, ubiquitination, and oligomerization, necrosulfonamide (NSA), could improve motor function and reduce dopaminergic neuron degeneration in a PD mouse model (Leem et al., 2023). Additionally, Nec-1s (Necrostatin-1s), a RIP signaling inhibitor, has been shown to increase the survival rate of TH-positive dopaminergic neurons in MPTP-induced PD mouse models, preventing behavioral, biochemical, and neurochemical alterations in the mice (Kartik et al., 2023). Lin et al. (2020) found that pre-treatment with Nec-1 or knockout of the RIPK3/MLKL genes in MPTP-treated mice led to increased levels of RIPK1, RIPK3, and MLKL proteins in the midbrain, which prevented dopaminergic (DA) neuron loss and reduced inflammation. Oliveira et al. (2021) found that necroptosis inhibitors exhibited protective effects on dopaminergic neurons in the substantia nigra and striatum in mice exposed to MPTP and further treated with Oxa12. Iannielli et al. (2018) used Nec-1 and Nec-1s in MPTP-induced Parkinson’s disease mouse models, which inhibited RIPK1, reduced dopaminergic (DA) neuron loss in the substantia nigra compacta (SNc), and prevented the decline in fiber density in the striatum.

Necroptosis is likely a critical step leading to the eventual death of these neurons, and this conclusion is well-supported by logical evidence. Firstly, evidence suggests that necroptosis is activated when apoptosis signaling is inhibited (Fritsch et al., 2019; Hartmann et al., 2001). This implies that during the process of cell death, if the apoptotic pathway is blocked, necroptosis may be initiated as an alternative pathway. Rotenone has been shown to reduce ATP levels in N2A cells, and a decrease in ATP levels generally inhibits apoptosis (Li et al., 2003). In experiments, N2A cells treated with rotenone showed a significant reduction in intracellular ATP content, along with an increase in necroptosis markers (Roy et al., 2023). This further suggests that the cells may undergo programmed cell death via the necroptosis pathway. Based on the above points, it can be inferred that during the onset and progression of PD, dopaminergic neuron death may initially involve factors that inhibit the apoptotic pathway, subsequently activating necroptosis. Thus, necroptosis becomes the final step in neuronal death.

### 3.2 α-Syn and necroptosis

#### 3.2.1 Aggregation and spread of α-Syn

Currently, all studies on the pathological mechanisms of PD are based on acute animal or cell models. It must be noted that these models have significant limitations; they lack predictive value for identifying neurodegenerative or neuroprotective agents and fail to successfully replicate key features of Parkinson’s disease progression, particularly the presence of Lewy bodies and Lewy neurites (Dawson et al., 2010). Lewy bodies (LBs) are important pathological markers of PD, primarily composed of fibrillar α-Syn, which severely disrupts normal cellular processes, leading to the death of dopaminergic neurons in the substantia nigra (SN) (Ahanger and Dar, 2024; Yan et al., 2024). Both sporadic and familial Parkinson’s disease patients share the characteristic presence of Lewy bodies in the brain (Schlossmacher et al., 2002; Spillantini et al., 1997; Spillantini et al., 1998). Aggregates of α-Syn constitute the core of Lewy bodies (LBs), and the aggregation of α-Syn is widely considered another major pathological feature of PD. Studies have shown a strong positive correlation between α-Syn aggregation and the loss of dopaminergic neurons in the substantia nigra (SN) (Braak et al., 2003; Dijkstra et al., 2014).

The normal clearance of α-Syn mainly depends on the ubiquitin-proteasome system (UPS) and the autophagy-lysosome pathway (ALP). However, under pathological conditions, these two key pathways are often impaired, leading to the accumulation of α-Syn (Saramowicz et al., 2023).

In PD, its pathogenic genes (*SNCA* :Synuclein Alpha, *LRRK2*: Leucine-Rich Repeat Kinase 2, *PRKN*: Parkin, and *PINK1*: PTEN-induced kinase 1) and risk gene (*GBA1*: Glucocerebrosidase 1) are closely associated with the accumulation of α-Syn (Funayama et al., 2023). First, regarding *SNCA*, mutations in this gene strongly promote α-Syn production, aggregation, and fibril formation (Bussell and Eliezer, 2004). This effectively adds a heavy burden on the degradation pathway of α-Syn, as increased production requires higher degradation capacity. Next is *LRRK2*, where mutations affect kinase activity, leading to abnormal protein phosphorylation and aggregation (Kim et al., 2020). This aggregation has multiple negative consequences: it exacerbates α-Syn-induced pro-inflammatory responses and neurotoxicity, induces oxidative stress, and promotes the formation of new aggregates in previously unaffected neurons (Kim et al., 2020; More et al., 2013; Tapias et al., 2017). These aggregates overload lysosomal function, ultimately worsening α-Syn accumulation. In addition, *LRRK2* mutations can affect mitochondrial homeostasis and enhance necroptosis (Weindel et al., 2022). The *PRKN* and *PINK1* genes function to eliminate dysfunctional mitochondria through specific mechanisms, thereby maintaining the quality of the organelle network (Narendra et al., 2008). When these two genes are mutated, mitophagy is impaired (Rakovic et al., 2019). It is important to note that mitochondrial quality control is achieved through the coordinated action of multiple mechanisms at different levels, including proteases, chaperones, the ubiquitin-proteasome system (UPS), and mitophagy (Vigié and Camougrand, 2017). Therefore, mutations in *PRKN* and *PINK1* may overload the UPS, leading to α-Syn accumulation. Finally, mutations in the risk gene *GBA1* can lead to lysosomal dysfunction and overload of the ubiquitin-proteasome system (UPS) (Rubilar et al., 2024). This not only impairs α-Syn degradation but also creates a vicious cycle where α-Syn accumulation further exacerbates lysosomal and proteasomal dysfunction.

In cases of PD, a small proportion is related to genetic factors, while the majority are identified as sporadic, with unknown causes (Funayama et al., 2023). However, mechanisms involving oxidative stress and mitochondrial dysfunction have been well established (Subramaniam and Chesselet, 2013). Considering multiple factors, it seems reasonable to define PD as an α-synucleinopathy. Factors such as pathogen-induced intestinal inflammation can lead to the local accumulation and aggregation of α-Syn in the enteric nervous system (Stolzenberg et al., 2017). Emerging evidence from laboratory models strongly suggests that aggregated forms of α-Syn can self-amplify and propagate in a “prion-like” manner through interconnected neural networks, following stereotypic and topographical patterns from peripheral tissues to the brain (Visanji et al., 2013). This provides an important clue regarding the etiology of sporadic PD cases. Given this, studying the impact of α-Syn aggregation on dopaminergic neuron death is crucial for gaining deeper insights into the pathological mechanisms involved in PD. First, from the perspective of the propagation mechanism, a study found that after PFF injection in transgenic A53T SynGFP mice, Lewy pathology spread retrogradely within neurons along the axon and trans-synaptically between neurons, extending from the peripheral nervous system to the central nervous system (Schaser et al., 2020). This phenomenon clearly demonstrates that in specific mouse models, the abnormal aggregation and propagation of α-Syn follow distinct pathways and directions. The discovery of this propagation pattern provides important clues for understanding the progression of PD, suggesting that the aggregation and propagation of α-Syn may be key factors in the advancement of the disease. Secondly, from the perspective of its impact on the nervous system, another mouse study found that injecting α-Syn PFFs (α-Syn preformed fibrils) into the striatum induced pathological aggregation of endogenous α-Syn. This led to a progressive loss of dopaminergic innervation and function in the striatum, as well as a slight reduction in synaptic density, accompanied by a neuroinflammatory response (Thomsen et al., 2021). This clearly demonstrates that the aggregation of α-Syn negatively affects multiple aspects of the mouse nervous system, including dopaminergic function, synaptic density, and immune response. These changes show a certain similarity to the clinical manifestations of PD patients, further confirming the significance of α-Syn aggregation in the pathological mechanisms of PD.

#### 3.2.2 Interaction between α-Syn aggregation and necroptosis

Experimental evidence has shown that carbon disulfide (CS_2_) can induce abnormal accumulation of α-Syn, which triggers necroptosis in dopaminergic neurons of the rat midbrain, ultimately resulting in Parkinsonian-like behavior in rats. In this study, through a series of rigorous experiments, it was strongly demonstrated that α-Syn interacts with necrosomes *in vivo*, *in vitro*, and in computer simulations, and this interaction is closely related to CS_2_ exposure. Furthermore, the study also found that the α-Syn inhibitor ELN484228 plays an important role. It significantly reduces CS_2_-induced α-Syn aggregation/phosphorylation and the activation of necroptosis signaling, thereby effectively decreasing cell loss (Liu et al., 2023). These findings delineate a close and significant relationship between α-Syn and necroptosis, suggesting that α-Syn may be pivotal in the initiation or progression of necroptosis. Recent investigations by Geng et al. (2023) provide additional support for this relationship, revealing that the deficiency of the necroptosis effector protein MLKL mitigates neuroinflammation and motor dysfunction in an α-Syn transgenic mouse model of Parkinson’s disease. Additionally, rapid oligomerization of α-Syn upon contact with the mitochondrial membrane was observed in induced pluripotent stem cell (iPSC)-derived neurons harboring A53T mutations from patients with Parkinson’s disease (PD), which can lower the threshold for mPTP opening and induce mitochondrial permeability transition pore opening (Choi et al., 2022). The activation of mPTP opening by α-Syn may be associated with augmented calcium release from endoplasmic reticulum-mitochondrial contact sites (MERCs), leading to mitochondrial matrix Ca^2+^ overload, and the calcium ions overload also promotes further aggregation of α-Syn (Ramezani et al., 2023).

Callizot et al. (2019) used “dopaminergic toxins” (MPP+, 6-OHDA, and rotenone) to induce mitochondrial damage in primary cultures of rat midbrain neurons. They found that dopaminergic toxins induce dopaminergic cell death through different pathways (Callizot et al., 2019). Necroptosis, a regulated form of cell death, is centered on the RIPK1-RIPK3-MLKL signaling cascade, with mitochondria serving as both regulatory hubs and effector executors. Mitochondrial reactive oxygen species (mtROS) have been shown to facilitate the formation of the RIPK1-RIPK3 complex by activating autophosphorylation of RIPK1, thereby enhancing the phosphorylation and membrane pore-forming capacity of MLKL and inducing necroptosis (Zhou et al., 2024). For example, in an ischemic stroke model, mtROS bursts resulting from mitochondrial dysfunction dramatically exacerbated necroptosis (Ren et al., 2023). Furthermore, RIPK3 activation can perturb mitochondrial energy metabolism and mtROS production, amplifying necrotic signaling (Li et al., 2024). Additionally, PGAM5, an atypical mitochondrial serine/threonine phosphatase, is implicated in mitochondrial autophagy and necrotic apoptosis. During severe mitochondrial stress and damage, PGAM5 elevates cyclophilin D phosphorylation levels and induces the opening of the mitochondrial membrane permeability transition pore (mPTP), triggering necroptosis (Zhou et al., 2018).

**FIGURE 2:**
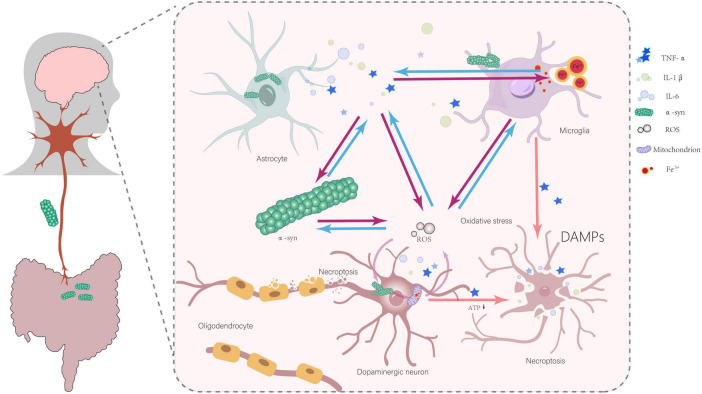
Abnormal α-Syn aggregation, inflammatory responses, iron deposition, mitochondrial dysfunction, oxidative stress, and interactions involving damage-associated molecular patterns (DAMPs) collectively contribute to the initiation and progression of necroptosis in dopaminergic neurons. All arrows indicate promoting effects.

Although the precise mechanism by which α-Syn affects the necroptosis remains to be elucidated (Liu et al., 2020), these studies highlight the undeniable close relationship between α-Syn and necroptosis. Combined with the aforementioned necroptosis-mediated axonal degeneration and the propagation and spread of α-Syn, this offers a highly valuable perspective for deepening our understanding of the pathogenesis of Parkinson’s disease from a new angle. In summary, research findings from various levels, including cell experiments and animal studies, collectively construct an important relationship map between α-Syn and necroptosis in the development of Parkinson’s disease (Figure 2).

α-Syn aggregation occupies a central role in the progression of Parkinson’s disease, with the process of inducing necroptosis in dopaminergic cells following a rigorous and complex logical sequence. α-Syn aggregation serves as the starting point for this entire chain reaction.

The activation of glial cells, the release of inflammatory cytokines, and the iron deposition in the substantia nigra pars compacta (SNpc) in the brains of PD patients have been well documented (Dexter et al., 1989; McGeer and McGeer, 2008; Yao et al., 2024). Studies have shown that abnormal aggregation of α-Syn can activate microglia and astrocytes, which in turn release large amounts of inflammatory cytokines, such as TNF-α, interleukin-1β (IL-1β), and interleukin-6 (IL-6) (Hughes et al., 2019; Kim et al., 2013). Among them, TNF-α has been clearly shown to be one of the key factors in inducing necroptosis in neuronal cells (Wang et al., 2022). The high sensitivity of oligodendrocytes to TNF-α and inflammatory signals may lead to myelin loss through necroptosis (Oñate et al., 2020; Yuan et al., 2019). In a study involving intranasal injection of α-Syn PFFs in cynomolgus monkeys, it was found that PFFs could induce iron deposition in microglia in the substantia nigra. This iron deposition may trigger neuroinflammation in the dopaminergic system (Guo et al., 2021). Excess iron also leads to the production of reactive oxygen species (ROS), increased oxidative stress levels in tissues, cellular damage, and ultimately cell death (Fischbacher et al., 2017). Another experiment using the ferroptosis inhibitor Fer-1 further demonstrated that it could inhibit ferroptosis in BV2 cells, downregulate the release of TNF-α in lipopolysaccharide-activated BV2 microglia, and reduce necroptosis in oligodendrocyte precursor cells (OPCs) (Chai et al., 2023). These pieces of evidence suggest the roles of iron deposition, inflammatory cytokines, and necroptosis in the pathological process of PD. Iron has been shown to promote α-Syn aggregation, while α-Syn aggregation can, in turn, affect iron metabolism. Additionally, iron promotes the inflammatory response of microglia (Johnson et al., 2019).

Furthermore, the NLRP3 inflammasome is a strong candidate of pathogenetic involvement and is regarded as a pivotal driver of PD-associated neuroinflammation (Wang et al., 2019). This is substantiated by the elevated gene expression of NLRP3, ASC, and CASP1, as well as the increased protein levels of NLRP3, cleaved caspase-1, and cleaved IL-1β in peripheral blood mononuclear cells sampled from PD patients, compared to healthy, age-matched controls (Fan et al., 2020). Additionally, NLRP3 is a well-established sensor of cytosolic mitochondrial DNA (mtDNA) and cardiolipin, which are released from mitochondria, and it also detects excessive mitochondrial reactive oxygen species (mtROS) production. In both hereditary and idiopathic forms of PD, microglial mitochondrial fission can trigger nuclear translocation of NF-κB and activation of the NLRP3 inflammasome, thereby intensifying neuroinflammation (Lawrence et al., 2022; Xu et al., 2023). Moreover, mitochondrial dysfunction induces a metabolic shift from oxidative phosphorylation (OXPHOS) to glycolysis in overactivated microglia, accompanied by an increased production of reactive oxygen species (ROS) and reactive nitrogen species (RNS). The excessive generation of ROS, culminating in oxidative stress, has emerged as a common underlying mechanism implicated in the chronic neuroinflammatory disorders characteristic of PD (Qin et al., 2024). Excessive activation of microglia leads to neurotoxicity and the production of large amounts of neuroimmune pro-inflammatory factors, which trigger oxidative stress and cause damage to dopaminergic neurons. A positive feedback loop exists between oxidative stress and inflammatory factors, forming a vicious cycle that further damages dopaminergic neurons and promotes disease progression (Morgan and Liu, 2011; Ouchi et al., 2009; Zhang et al., 2021). Moreover, the aggregation of α-Syn and the activation of glial cells, which produce inflammatory factors, have a bidirectional causal relationship. This leads to persistent inflammation and ongoing α-Syn aggregation, creating a vicious cycle (Kim et al., 2013; Lee et al., 2019; Wang et al., 2016). This series of processes clearly demonstrates that α-Syn aggregation, through triggering inflammatory responses, may ultimately lead to ongoing necroptosis of dopaminergic neurons.

Studies have definitively confirmed that abnormal aggregation of α-Syn can disrupt normal mitochondrial metabolism and function, leading to excessive production of reactive oxygen species (ROS) by mitochondria, which damages various cellular structures and functions (Tapias et al., 2017). The experimental results of Callizot et al. (2019) strongly support this view. They found that oxidative stress induces the death of neurons expressing tyrosine hydroxylase (TH) and promotes the formation of α-Syn aggregates, which are present both inside and outside of neurons (Callizot et al., 2019). This clearly indicates a mutually reinforcing relationship between oxidative stress and α-Syn aggregation. On one hand, α-Syn aggregation leads to mitochondrial dysfunction, which in turn increases oxidative stress. On the other hand, oxidative stress further promotes the aggregation of α-Syn, forming a vicious cycle that continuously exacerbates cellular damage. Extracellular α-Syn aggregates can activate microglia (Callizot et al., 2019). Once microglia are uncontrollably activated, they can directly impact neurons by releasing various inflammatory mediators, which in turn induce oxidative stress (More et al., 2013). This process leads to the formation of new α-Syn aggregates in unaffected neurons, thereby driving the progression of neurodegeneration (Callizot et al., 2019; More et al., 2013). This process forms a powerful feedback loop that continuously exacerbates neuroinflammation and α-Syn aggregation. In this feedback loop, α-Syn aggregation, iron deposition, microglial activation, inflammatory mediator release, and oxidative stress interact with each other, collectively driving the progression of Parkinson’s disease. It is known that reactive oxygen species (ROS) may regulate necroptotic signaling and cell death through various pathways (Fulda, 2016). In the context of increased intracellular oxidative stress caused by α-Syn aggregation, ROS levels also rise accordingly. These ROS may directly or indirectly act on intracellular signaling pathways, triggering the necroptosis signaling cascade, ultimately leading to necroptosis in dopaminergic cells. In addition, mitochondrial dysfunction caused by α-Syn aggregation leads to reduced ATP levels in the cell (Wilkaniec et al., 2021). Low ATP levels may promote the cell’s tendency to undergo necroptosis (Roy et al., 2023). α-Syn aggregation may trigger the release of inflammatory cytokines through various factors, including iron deposition, leading to neuronal damage and ultimately necroptosis. Necroptosis, in turn, can exacerbate inflammation through the release of damage-associated molecular patterns (DAMPs), forming a vicious feedback loop (Eigenbrod et al., 2008). The model proposed by Johnson et al. (2019) emphasizes that the death of dopaminergic neurons in Parkinson’s disease is more likely the result of multiple factors working in concert, with α-Syn aggregation playing a key role in this process.

In summary, α-Syn aggregation ultimately leads to necroptosis in dopaminergic neurons through a series of complex processes, including mitochondrial dysfunction, oxidative stress, microglial activation, and the formation of feedback loops, creating a complex pathological cycle. This logical relationship provides an important theoretical basis for understanding the pathogenesis of Parkinson’s disease and indicates potential therapeutic targets for developing new treatments.

## 4 A hypothetical model of space-time

**FIGURE 3:**
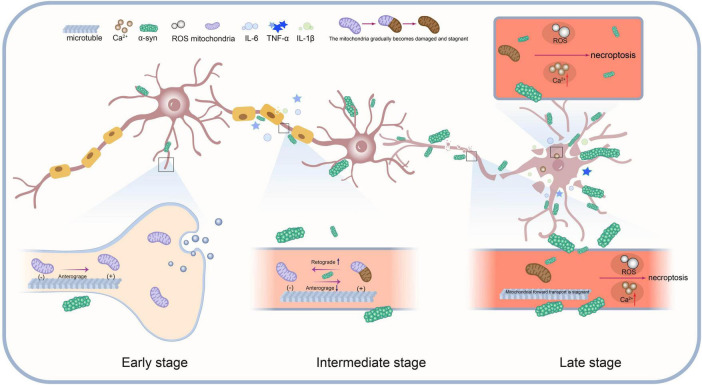
This model illustrates the dynamic interactions between mitochondrial transport impairment, α-Syn protein aggregation, neuroinflammation and necroptosis during the progression of Parkinson’s disease.

Many neuropathies are caused by axonal degeneration, which begins at the distal end of the diseased axon and propagates retrogradely toward the cell body, ultimately leading to neuronal death (Cavanagh, 1979; Coleman, 2005). Mitochondrial transport is also carried out along axons (Hollenbeck and Saxton, 2005; Sheng and Cai, 2012). The aggregation of α-Syn not only impairs normal mitochondrial function (Tapias et al., 2017) but may also directly interfere with mitochondrial transport in axons by disrupting transport-associated proteins and motors (Li et al., 2013). A spatiotemporal perspective can reveal how these region-specific changes, as well as cell-cell interactions, drive disease progression. Furthermore, given that Parkinson’s disease is a progressive condition, elucidation of its pathomechanisms in temporal dimensions may facilitate more precise comprehension of dynamics of disease progression and the distinctive characteristics of each stage. By associating it with the retrograde axonal transport of α-Syn mentioned earlier and integrating the classic Braak staging system, which explains PD pathology (Braak et al., 2003), we propose a spatiotemporal model of dopaminergic neurodegeneration in Parkinson’s disease (Figure 3):

(1) Early Stage: α-Syn aggregates in the synaptic region. Mitochondrial transport remains normal, but mild α-Syn aggregation begins to impair mitochondrial function.
(2) Intermediate Stage: α-Syn aggregation increases, worsening mitochondrial damage. Anterograde transport is progressively impaired, especially in synapses and axons. Damaged mitochondria stall and undergo retrograde transport for degradation or repair. Mitochondrial damage and α-Syn aggregation activate necroptosis signals, accelerating disease progression.
(3) Late Stage: Mitochondrial transport nearly halts, and α-Syn aggregates extensively within the cell. Oxidative stress and Ca^2+^ imbalance further promote necroptosis. Ultimately, cell death occurs via necroptosis pathways.
(4) Spatiotemporal Interaction: Mitochondrial transport, α-Syn aggregation, and necroptosis interact across time and regions. Synapse, axon, and cell body functions decline progressively, driving pathological spread.

This spatiotemporal model underscores the pivotal role of necroptosis in PD and elucidates its interaction with mitochondrial transport disruption and α-Syn aggregation. As the disease progresses, mitochondrial dysfunction and α-Syn accumulation intensify, initiating necroptosis. This process is further amplified by key factors such as the release of inflammatory mediators, forming a positive feedback loop that exacerbates necroptosis (Figure 2) ultimately driving neuronal death.

In addition to the spatiotemporal model we proposed, there are other hypothesis models for the pathological mechanisms of Parkinson’s disease (PD), each with its own advantages and limitations. For example, the early classical integrated model, which integrates molecular mechanisms such as mitochondrial dysfunction, oxidative stress, and α-Syn aggregation (Deng et al., 2018; Goedert, 2001; Nagatsu and Sawada, 2006; Polymeropoulos et al., 1997; Subramaniam and Chesselet, 2013; Wang and Youle, 2009), provides a basic explanatory framework for dopaminergic neuron degeneration. However, its static nature makes it difficult to explain the spatiotemporal dynamics of disease progression. The single-neuron degeneration model compensates for some of these shortcomings. This model successfully explains the subtle motor symptoms in the early stages of PD and the slow progression of the disease by proposing a chronic neuronal death mechanism mediated by endogenous neurotoxins, such as MPTP (Huenchuguala and Segura-Aguilar, 2024). However, both of these models still have significant limitations in explaining non-motor symptoms and the dynamic pathology spread. The spatiotemporal model emphasizes that the speed of PD progression is influenced by the α-Syn clearance mechanism. In the early stages, this clearance mechanism can delay α-Syn accumulation and pathological spread, which helps explain the subtle motor symptoms and slow progression seen in the early stages of PD. From the perspective of the spatiotemporal model, endogenous toxins are more like one of the factors that promote the abnormal aggregation of α-Syn and other pathological processes. Various mutually promoting pathological reactions lead to the occurrence of programmed neuronal cell death (Figure 2). Compared to the classical integrated model and the single-neuron degeneration model, the spatiotemporal model provides a more comprehensive and detailed explanatory framework, capable of explaining both motor and non-motor symptoms as well as the spatiotemporal dynamics of pathological progression. By integrating the phase characteristics of disease progression with dynamic changes in time and space, the model constructs a multidimensional analytical framework. It focuses on the differences in disease presentation at different stages and regions, revealing the interactions and evolutionary patterns of multiple pathological mechanisms at specific times and regions during each stage. By combining the diffusion pathways of the disease in Braak’s theory with the accumulation of pathological markers in each stage, it systematically explains how local lesions are related to overall disease development in time and space. This approach, which allows for a detailed analysis of temporal and spatial changes while coherently tracking the entire progression, provides a clearer perspective for understanding the disease and will also offer insights for the early diagnosis and treatment strategies of PD.

The following are treatment strategies designed according to the different pathological stages of PD:

(1) Early Stage: α-Syn aggregates in the synaptic region, mitochondrial transport is normal but mildly impaired. Treatment focuses on maintaining mitochondrial function and slowing α-Syn aggregation, using antioxidants, mitochondrial protectants, etc.
(2) Intermediate Stage: α-Syn aggregation and mitochondrial damage intensify, anterograde transport is obstructed, and necroptotic apoptosis signals are activated. Treatment should focus on improving mitochondrial transport and inhibiting necroptotic apoptosis through mitochondrial protectants, necroptotic inhibitors, etc.
(3) Late Stage: Mitochondrial transport almost ceases, and cells enter the necroptotic apoptosis process. The treatment goal is to prevent cell death, using necroptotic therapy, neuroprotective drugs, etc.
(4) Spatiotemporal Interaction: Different pathological processes interact, promoting pathological spread. Treatment should employ a multi-target strategy to slow down the overall progression of PD.

## 5 Conclusion

This review highlights the critical role of necroptosis in PD and its intricate interplay with α-Syn aggregation. Unlike traditional studies that primarily focus on apoptosis or autophagy, this review underscores necroptosis as a significant mechanism driving dopaminergic neuronal death. Bioinformatics analysis further supports this role, revealing that the necroptosis pathway is significantly activated in PD samples (Lin et al., 2023). Among the genes involved, 12 key necroptosis-related genes, such as ASGR2, CCNA1, and FGF10, are crucial in neuronal death and PD progression (Lei et al., 2023). The abnormal expression of these genes likely contributes to the activation of the necroptosis pathway, collectively influencing the course of the disease.

A key innovation of this work is the proposed spatiotemporal model of PD progression, which integrates α-Syn aggregation, mitochondrial dysfunction, oxidative stress, neuroinflammation, and necroptosis into a unified framework. This model explains how early α-Syn aggregation impairs mitochondrial function and transport, initiating necroptosis through oxidative stress and inflammatory responses. These pathological processes are mutually reinforcing, forming a vicious cycle that exacerbates neuronal degeneration and facilitates disease progression. This model offers a novel perspective on understanding the pathological progression of PD, particularly in terms of the connection between different disease stages and clinical symptoms (such as early non-motor symptoms and later motor symptoms). However, despite the reasonable hypotheses derived from existing literature and theoretical frameworks, further experimental validation is needed. Future research should focus on several key areas: First, using animal models and cell experiments to validate the interactions of various pathological factors in the spatiotemporal model and their temporal changes across different disease stages. Second, investigating the effects of necroptosis inhibitors on this model to assess their potential in delaying neurodegeneration, reducing oxidative stress, and mitigating inflammation. Additionally, as clinical data accumulates, further validation of the model’s potential in early non-motor symptom identification and therapeutic intervention is necessary. The spatiotemporal model not only provides a new theoretical framework for the pathological mechanisms of PD but also guides future experimental research and clinical applications. With deeper experimental validation, the spatiotemporal model holds promise as an important tool for early diagnosis, disease progression assessment, and therapeutic intervention, potentially driving the development of more effective treatment strategies.

In summary, this review integrates current findings to present necroptosis as a central mechanism in PD pathogenesis, while offering novel perspectives on its interaction with α-Syn aggregation. By proposing a spatiotemporal model and discussing the therapeutic potential of necroptosis inhibition, this work lays the foundation for future research and clinical strategies aimed at developing more effective interventions for PD.
